# Zeroth- and first-order long range non-diffracting Gauss–Bessel beams generated by annihilating multiple-charged optical vortices

**DOI:** 10.1038/s41598-020-78613-7

**Published:** 2020-12-15

**Authors:** Lyubomir Stoyanov, Maya Zhekova, Aleksander Stefanov, Ivan Stefanov, Gerhard G. Paulus, Alexander Dreischuh

**Affiliations:** 1 Department of Quantum Electronics, Faculty of Physics, Sofia University, 5, J. Bourchier Blvd., Sofia, 1164 Bulgaria; 2 Department of Mechatronics, Robotics and Mechanics, Faculty of Mathematics and Informatics, Sofia University, 3, J. Bourchier Blvd., Sofia, 1164 Bulgaria; 3 Institute of Mathematics and Informatics, Bulgarian Academy of Sciences, Acad. Georgi Bonchev Str., Block 8, Sofia, 1113 Bulgaria; 4 Institute of Optics and Quantum Electronics, Friedrich Schiller University, Max-Wien-Platz 1, 07743 Jena, Germany; 5 Helmholtz Institute Jena, Helmholtzweg 4, 07743 Jena, Germany

**Keywords:** Optics and photonics, Optical physics, Optical techniques, Transformation optics

## Abstract

We demonstrate an alternative approach for generating zeroth- and first-order long range non-diffracting Gauss–Bessel beams (GBBs). Starting from a Gaussian beam, the key point is the creation of a bright ring-shaped beam with a large radius-to-width ratio, which is subsequently Fourier-transformed by a thin lens. The phase profile required for creating zeroth-order GBBs is flat and helical for first-order GBBs with unit topological charge (TC). Both the ring-shaped beam and the required phase profile can be realized by creating highly charged optical vortices by a spatial light modulator and annihilating them by using a second modulator of the same type. The generated long-range GBBs are proven to have negligible transverse evolution up to 2 m and can be regarded as non-diffracting. The influences of the charge state of the TCs, the propagation distance behind the focusing lens, and the GBB profiles on the relative intensities of the peak/rings are discussed. The method is much more efficient as compared to this using annular slits in the back focal plane of lenses. Moreover, at large propagation distances the quality of the generated GBBs significantly surpasses this of GBBs created by low angle axicons. The developed analytical model reproduces the experimental data. The presented method is flexible, easily realizable by using a spatial light modulator, does not require any special optical elements and, thus, is accessible in many laboratories.

## Introduction

More than 30 years ago, the first non-singular solution of the scalar Helmholtz wave equation describing non-diffracting beams, namely the zeroth-order Bessel function of the first kind, was reported by Durnin^1^. More generally, plane waves (in rectangular coordinates), Bessel beams (in circular cylindrical coordinates), Mathieu beams (in elliptic cylindrical coordinates), and parabolic beams (in parabolic cylindrical coordinates) are exact solutions of the Helmholtz equation (see^2^ and the references therein). In the review paper of Bouchal^3^, the physical concept of the non-diffracting propagation is presented and the basic properties of the non-diffracting beams are reviewed. It is very intriguing to follow the early history and discussion regarding the term “non-diffracting” beams^4^—beams whose central maxima are remarkably resistant to diffractive spreading commonly associated with all wave propagation. Mathematically, the Bessel beam has an infinite number of rings, and hence, it is carrying infinite power/energy and cannot be generated in the exact sense. However, Durnin et al. showed that one can generate its reasonable approximation^5^. They also showed^6^ that the depth-of-field of a Bessel beam can be made arbitrarily larger than that of a Gaussian beam having the same spot size. Characteristic of the phase fronts of zeroth-order Bessel beams are the $\pi$-phase shifts from one ring to the next and the planar phase front of the central peak as shown interferometrically in Ref.^7^.

The Bessel beam can be viewed as a superposition of plane waves whose wavevector is directed along a cone with respect to the propagation axis. Hence, in a natural way, the idea to generate Bessel beams (BBs) by using axicons arises^4^. In the terahertz region, a scheme for phase manipulation based on parallel-plate waveguides was shown to reproduce the functionality of an axicon^8^. Segmented deformable mirrors creating the phase structure of a reflective axicon were shown to be applicable for the generation of zeroth- and higher-order BBs with controllable transverse and longitudinal shapes^9^. Integrated optical phased arrays, using a one-dimensional splitter with a tree-based phased array architecture were also demonstrated^10^. In a certain sense, this technique can be viewed as an alternative realization of the above mentioned approaches.

By implementing a computer-generated hologram of an axicon using a spatial light modulator (SLM) as a diffractive optical element^11^ and then combining it with a prism to compensate for the dispersion, white-light achromatic Bessel beams were generated^12^. An alternative approach based on the use of an SLM and an iterative Fourier transformation algorithm was also demonstrated^13^. Even arrays of Bessel beam can be generated using a SLM directly introducing in the modulator the phase variation of an axicon^14^. Since the angular spectrum of the Bessel function is a ring, one can think to generate such beams by placing an annular slit in the back focal plane of a lens^4,5,15^. Instead of using an annular slit, efficient Bessel beam generation can be achieved by lasers with unstable cavities generating ring-shaped beams^7^, eventually adding a hard aperture to slightly reshape the outer ring diameter only. Gauss–Bessel beams can also be generated by using computer-generated holograms^16^ and, as recently shown for the 10-GHz band only, by using polarization insensitive metasurfaces^17^.

One more work is worth mentioning in relation to the results in this paper. Generation of Bessel beams in free space reaching, to the best of our knowledge, diffraction-free propagation distances of several meters at optical wavelength, as reported in Ref.^18^. The key optical component used, is a ring-lens which resembles a cylindrical lens that is morphed to a closed ring-like form. The authors comment correctly that, in a certain sense, their approach is similar to the annular slit used by Durnin and co-workers^5^, however, without the losses introduced by the annular aperture.

The interest for researchers in non-diffracting beams roots in applications spanning from secure sharing cryptographic keys^19^ to propagation in turbulence^20^. It is also motivated by their ability to reconstruct their initial amplitude profile in free-space propagation after being disturbed by an obstacle^21^. In all-optical photoacoustic microscopy the use of BBs leads to an increase in the depth of focus by a factor of ten^22^ as compared to the case when a Gaussian beam is used. More recent applications include real-world free-space optical communications (outdoor optical link of 150 m^23^), (de)multiplexing vortex modes^24^, tomography with BBs^25,26^, nonlinear propagation for creating filaments in transparent media^27^, and even high-order harmonic generation^28^. Last but not least, without any claim of completeness, BBs are used for precision femtosecond laser micromachining (e.g. high aspect ratio (length/diameter) nanochannel machining, photopolymerization, nanopatterning, processing of uneven surfaces, etc.)^29–34^, for optical trapping and tweezing^35,36^, for optical microscopy^37^, and even for laser particle acceleration^38,39^. Perfect optical vortices with ring sizes that do not depend on their topological charges, are generated by Fourier-transforming Bessel beams^40,41^. In the nonlinear optics, the dynamics of optical vortices nested in Bessel beams and propagating in third-order nonlinear media with noticeable nonlinear absorption was found to strongly contrast with their behavior in usual Gaussian-like beams^42,43^. The existence of Bessel–Bessel light bullets with complicated intensity distributions is proposed in^44^.

In this work, we demonstrate a novel approach for generating zeroth- and first-order long-range non-diffracting Gauss-Bessel beams (GBBs). As in Refs.^5,18^, the key point is the creation of a bright ring-shaped beam with a large ring radius-to-width ratio, which is subsequently Fourier-transformed by a thin lens. However, in contrast to previous works, our approach is entirely based on linear singular optics. We generate the required ring-shaped beam by creating an optical vortex with a very high topological charge and subsequently annihilating these (or one less) before performing the Fourier transformation into the GBB with a thin lens. The method is much more efficient as compared to using annular slits in the back focal plane of lenses. It is shown that at large propagation distances, the quality of the generated GBBs significantly surpasses this of GBBs created by low angle axicons. The approach is easy to apply, can be seamlessly realized using a single SLM, and can generate higher-order GBBs.

## Analytical model

For a theoretical analysis of our idea, we adapt the approach described in Ref.^40^ to perfect optical vortices. Let us denote by $F\{U\}$ the Fourier transform of an axial-symmetric function $U(x,y)=U(r,\theta )$ where (*x*, *y*) and $(r,\theta )$ are the transverse Cartesian and polar coordinates, respectively. Further, $H_n \{R(r)\}$ denotes the Hankel transform of order *n* of a function *R*(*r*). The spatial frequencies corresponding to coordinates (*x*, *y*) and $(r,\theta )$ will be denoted by $(f_x,f_y)$ and $(\rho ,\varphi )$. $U(r,\theta )$ is supposed to be a function separable in polar coordinates, $U(r,\theta )=R(r)V(\theta )$. According to^45^

1 $$\begin{aligned} F\{U(r,\theta )\} = \sum _{n=-\infty }^{\infty } c_n (-i)^n \exp (in\varphi ) H_n\{R(r)\} , \end{aligned}$$

where

2 $$\begin{aligned} c_n=\frac{1}{2\pi }\int _{0}^{2\pi }V(\theta )\exp (-in\theta ) d\theta . \end{aligned}$$

In the case of $V(\theta )=\exp (il\theta )$, where *l* is an integer, the coefficient $c_n=1$ for $n=l$ and $c_n=0$ for $n \ne l$. Accordingly, the Fourier transform of Eq. (1) is

3 $$\begin{aligned} F\{U(r,\theta )\} = (-i)^l \exp (il\varphi ) H_l\{ R(r) \} . \end{aligned}$$

**Figure 1:**
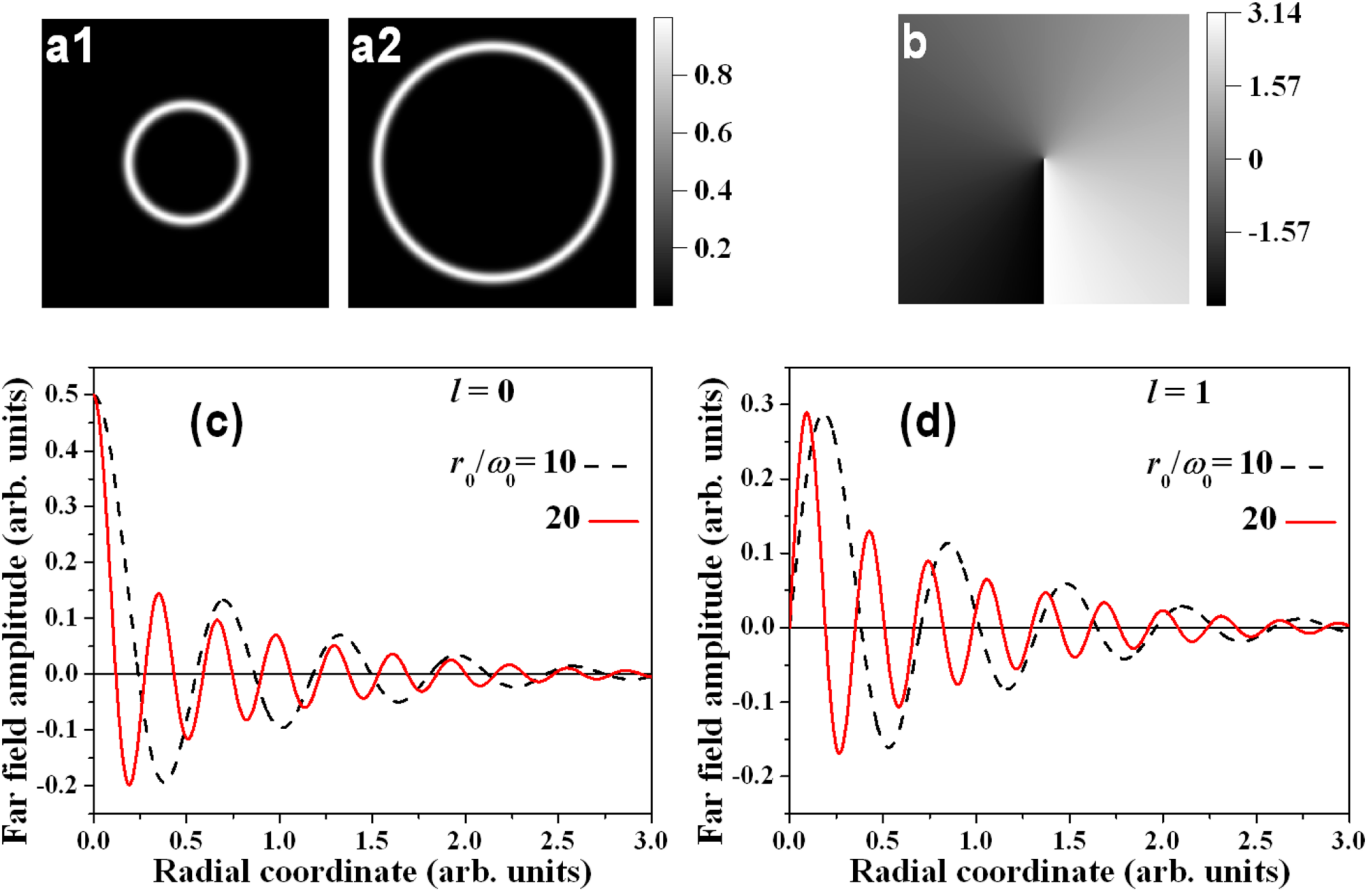
Distribution of the *input* electric field amplitude *E* of the light beam described by Eq. (6) for $r_0/\omega _0=10$ (panel (**a1**)) and 20 (panel (**a2**)), where $\omega _0$=const. Panel (**b**)—typical phase profile of the central peak of the first-order GBB. Radial distributions according to Eq. (10) of *the far field* electric field amplitudes $E'$ of zeroth- (**c**) and first-order GBBs (**d**) for $r_0/\omega _0$=10 (black dashed curves) and 20 (red solid curves).

Applying the Fourier transform twice we get

4 $$\begin{aligned} F^2\{U(r,\theta )\} = (-i)^{2l} \exp (il\varphi ) H_l^2\{ R(r) \} = (-i)^l U(r,\theta ) , \end{aligned}$$

since the Hankel transform is its own inverse^46^. Another important identity we need is (p. 622, 6.633, f2 in^47^)

5 $$\begin{aligned} \exp \bigg (-\frac{r^2 + r_0^2}{\omega _0^2}\bigg )I_l\bigg (\frac{2rr_0}{\omega _0^2}\bigg ) = \frac{\omega _0^2}{2}\int _0^\infty \exp \bigg (-\frac{\omega _0^2\rho ^2}{4} \bigg ) J_l(r_0\rho )J_l(r\rho )\rho d\rho = \frac{\omega _0^2}{2} H_l \bigg \{ \exp \bigg (-\frac{\omega _0^2\rho ^2}{4} \bigg ) J_l(r_0\rho ) \bigg \} , \end{aligned}$$

where $I_l(...)$ is the modified Bessel function of the first kind and $J_l(...)$ is the Bessel function, both of order *l*. The electric field amplitude *E* of the light beam is described by

6 $$\begin{aligned} E(r,\theta ) = \exp (il\theta ) \exp \biggl \{-\frac{(r-r_0)^2}{\omega _0^2} \bigg \} = \exp (il\theta ) \exp \bigg \{-\frac{r^2+r_0^2}{\omega _0^2}\bigg \} \exp \bigg \{\frac{2rr_0}{\omega _0^2}\bigg \} , \end{aligned}$$

where $r_0$ and $\omega _0$ are positive real parameters denoting the radius of the bright vortex ring and the ring width, respectively (see Fig. 1(a1,a2)). When $r_0 \gg \omega _0$, the last exponent in Eq. (6) can be viewed as an approximation of the modified Bessel function of the first kind

7 $$\begin{aligned} \exp \bigg \{\frac{2rr_0}{\omega _0^2}\bigg \} \approx I_l\bigg \{\frac{2rr_0}{\omega _0^2}\bigg \} , \end{aligned}$$

which means that

8 $$\begin{aligned} E(r,\theta ) = \exp (il\theta ) \exp \bigg \{-\frac{r^2+r_0^2}{\omega _0^2}\bigg \} I_l \bigg \{\frac{2rr_0}{\omega _0^2}\bigg \} . \end{aligned}$$

Applying the results given by Eqs. (5) and (3) for the electric field amplitude, we get

9 $$\begin{aligned} E(r,\theta ) = \frac{\omega _0^2}{2} \exp (il\theta ) H_l \bigg \{ \exp \bigg ( -\frac{\omega _0^2 \rho ^2}{4} \bigg ) J_l(r_0\rho ) \bigg \} = \frac{\omega _0^2}{2(-i)^l} F\bigg \{ \exp (il\theta ) \exp \bigg ( -\frac{\omega _0^2 \rho ^2}{4} \bigg ) J_l(r_0\rho ) \bigg \} . \end{aligned}$$

By taking the Fourier transform again and considering Eq. (4), we obtain

10 $$\begin{aligned} E'(\rho ,\varphi ) = \frac{\omega _0^2}{2} \exp \{il(\varphi -\frac{\pi }{2}) \} \exp \bigg (-\frac{\omega _0^2\rho ^2}{4}\bigg )J_l(r_0\rho ) \end{aligned}$$

for the electric field amplitude $E'$ of the optical beam in the far field. From this equation it is evident that the last two multipliers describe a Gauss–Bessel beam of order *l*. The phase term in the first exponent provides the desired physical insight that this order *l* has the meaning of the residual topological charge of the optical vortex (OV). In view of the preceding assumptions, the bright amplitude/intensity ring of radius $r_0$ has to be much larger than the ring width $\omega _0$. In this paper, we will further concentrate on the cases $l=0$ and 1, although higher-order GBBs can be generated by higher residual topological charges *l*.

In panels (a1) and (a2) of Fig. 1 we show the *input* amplitude profiles of bright vortex rings calculated using Eq. (6) for $r_0/\omega _0$ = 10 and 20. When the residual $l=0$, the phase front of the beam is flat and we forgo showing it and rather only display the spiral phase profile of the central peak of the first-order GBB carrying an optical vortex with a residual $l=1$ (see Fig. 1(b)). In panel (c) of Fig. 1, numerically-calculated (see Eq. (10)) cross-sections of the *far-field* amplitude profile of zeroth-order GBBs with $r_0/\omega _0$ = 10 and 20 are shown. The smaller input ring shown in frame (a1) results in a GBB with a wider central peak and larger radii of the surrounding rings after the Fourier-transformation. The same tendency is clearly seen in Fig. 1(d) for first-order GBBs. However, the presence of a point phase dislocation (OV) on the beam axis causes a central ring instead of a peak.

## Experimental results

**Figure 2:**
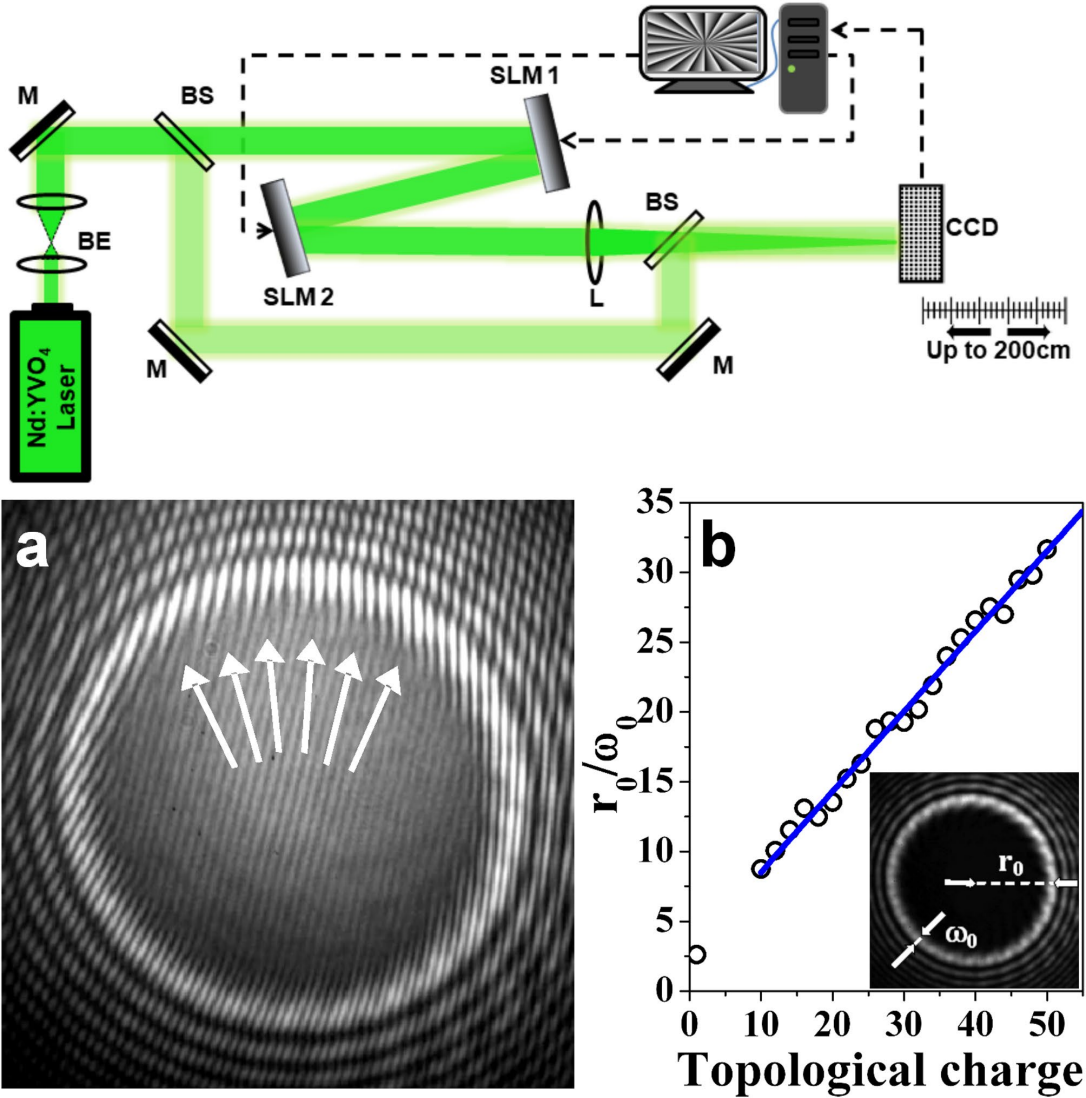
Top—experimental setup. Nd:YVO$_4$, continuous-wave frequency-doubled solid-state laser emitting at a wavelength $\lambda$=532 nm; BS, beam splitters; M, flat silver mirrors; SLM, reflective spatial light modulators (model Pluto, Holoeye Photonics); L, focusing lens (diameter 2.5 cm, *f*=100 cm); CCD, charge-coupled device camera. Bottom: **(a)** Magnified interference pattern recorded in front of the lens when SLM1 is programmed to imprint on the flat phase front of the incoming Gaussian beam the helical phase of an optical vortex with a topological charge 25. SLM2 is switched off. Arrows point to the positions of 6 from the 25 decayed OVs. **(b)** Dependence of the ring radius-to-width ratio $r_0/\omega _0$ on the topological charge encoded by one of the SLMs at a fixed distance of 50 cm behind it. Inset (shrunken): Intensity distribution of the bright vortex ring shown in panel **(a)** when the reference arm of the interferometer is blocked, with designation of $r_0$ and $\omega _0$.

The experimental setup used is shown in the upper part of Fig. 2. It involves a 532 nm continuous-wave beam from a frequency-doubled Neodymium-doped yttrium orthovanadate (Nd:YVO$_4$) laser and two reflective liquid-crystal spatial light modulators SLM1 and SLM2 of the same type (1920 pix.$\times$1080 pix.; pixel pitch $- 8\,\upmu m$; 4.5$\pi$ phase shift at 532 nm; 82% reflectivity). The Gaussian laser beam (2.6 mm FWHM; M$^2$-factor 1.2) illuminates SLM1. On this first modulator we project the phase of the desired highly-charged OV as illustrated on the screen of the computer sketched in Fig. 2 (top). SLM1 modifies the phase of the input Gaussian beam (and, as a consequence, also its amplitude/intensity) and redirects it to a second spatial light modulator SLM2 of the same type. On SLM2 we project the phase of the same highly-charged OV (for subsequent zeroth-order GBB generation) or phase with one unit TC more/less in order to obtain a residual TC $l=\pm 1$ (for subsequent first-order GBB creation). It is worth mentioning that because of the reflection from SLM2 the signs of the TCs of the encoded OVs are effectively opposite to the signs of OVs created by SLM1. As a result, in the plane of the focusing lens, in the considered cases the residual TCs are zero or $\pm 1$. The double modulated singular beam reflected from SLM2 is Fourier-transformed by a lens L (diameter 2.5 cm, $f=100$ cm). The CCD camera (chip sensitive area 7.1 mm$\times$5.4 mm; 1600 pix.$\times$1200 pix.) was carefully adjusted in and up to 2 m behind its focus. In order to do this, the CCD camera is placed on a rail such that it can controllably be translated along the beam axis. The distance between SLM2 and the lens is 95 cm. This distance is short enough to fit the ring-shaped beam with the largest radius $r_0$ (see Fig. S1) into the central part of the lens’ aperture. A reference beam is split off the laser beam before SLM1 by a beam splitter (BS). The object and the reference beams are recombined by a second BS to interfere on the CCD camera chip. Power density distributions and interference patterns are recorded by blocking/unblocking the reference laser beam. SLM1 and SLM2 are aligned parallel to each other with a distance of 49 cm. The angle of incidence of the green laser beam is $\sim 4^\circ$. When switched off, each SLM is acting as a flat mirror only.

Intuitively, in an optical setup involving two SLMs, one should expect that a lens has to be used in order to eliminate the effect of diffraction. In our case, however, we need the diffraction in order to allow the multiple-charged OV reflected from SLM1 to decay in to singly-charged OVs. Decay means splitting of OV with a TC $|m|>$1 into |*m*| single-charged OVs^48^ in the presence of even small coherent background beam. Such coherent background beam is presented in the generated singular beams at least due to the incoming beam diffraction by the SLM active area structured in pixels. It is known that the OVs behave in some degree as charged particles^49^. They may rotate around the beam axis, repel, attract each other, and annihilate in collision^48,50,51^. The needed broad ring-shaped beam in front of SLM2 is formed as a result of the OV mutual repulsion^49^ for which a certain propagation length is needed. For the same reason, we need some propagation length from SLM2 to the focusing lens L ( see Fig. S1 in the Supplementary material). Since we project a multiple-charged OV on SLM2, its decay into a ring of singly-charged vortices is important for the annihilation of the OVs generated by the first SLM before the focusing lens is reached.

In the lower left panel (a) in Fig. 2 we show an interference pattern recorded in front of the focusing lens L, when SLM1 is programmed to imprint the helical phase of an optical vortex with a topological charge 25 on the incoming Gaussian beam. SLM2 is then switched off. The intensity distribution of the bright vortex ring when the reference arm of the interferometer is blocked, is shown (shrunken) as an inset in the graph in panel (b). The influence of the diffraction on the bright vortex ring is evident as radial oscillating rings around the singular beam. These side lobes can effectively be suppressed when an annular phase plate of suitable inner and outer radii is used (see e.g. Fig. 4 in^52^). Closer inspection of the interferogram in panel (a) of Fig. 2 provides interesting information. Since OV with high TC tends to be unstable^48^, it comes as no surprise that one can easily find 25 singly-charged OVs by exploring the inner arc of the bright ring. The presence of these decayed singly-charged OVs is evident by the white arrows pointing to the fork-shaped splittings of the interference lines. All fork-shaped splittings have the same direction indicating that all 25 singly-charged OVs are with the same sign. Repelling each other and reordering themselves^49^, they appear arranged near the inner arc of the OV ring. In the middle there are no vortices. That is why some light is present in the central area of this dark beam and parallel interference lines are clearly seen. Another important observation in the context of the presented theoretical model is that in the case of a $|TC|=25$ the ring radius-to-width ratio $r_0/\omega _0\approx 16.7$ i.e. it is much larger than unity and thus fulfills the requirement of our theoretical model. This value was extracted from the inset in Fig. 2(b). The particular values of the ring radii $r_0$ are measured in diametrical slices, whereas the ring widths $\omega _0$ correspond to radial half-widths of the respective bright arcs at 1/*e* levels. For clarity of presentation, we denote $r_0$ and $\omega _0$ in the inset in Fig. 2(b). The OV TC is the important parameter up to the reflection from SLM2. Thereafter, and until the focusing lens is reached, the major beam parameter characterizing the GBB formation and propagation is the ring radius-to-width ratio $r_0/\omega _0$. In the described experimental setup, as illustrated on the graph in Fig. 2(b), the ring radius-to-width ratio $r_0/\omega _0$ increases linearly with increasing the OV TC, although the growth rate can be expected to depend on the used device (mode convertor or SLM; see e.g.^53,54^ and the references therein). In our case, for TCs between 2 and 6, we always observed decay of the multiple-charged vortex state into singly-charged OVs leading to an irregular modulation of the background Gaussian beam. Well formed bright OV rings, however, were observed for $|TC|\ge 10$.

At this point, we have seen that highly charged vortices can provide ring-shaped bright beams reminiscent of those required for the generation of GBBs. However, they still contain many phase singularities which need to be removed. In order to see how this can be accomplished, one should recall (see the experimental setup in Fig. 2) that we have the degree of freedom to program the second spatial light modulator SLM2 as well. Here an important comment has to be made. The phase profiles we send to the SLMs are those of a multiple charged OV, which can be viewed as many singly charged OVs with precisely aligned axes and cores. This multiple charged OV decays into single charged vortices which repel and rearrange as shown in the interferogram in Fig. 2(a). For the creation of a zeroth-order Gauss-Bessel beam in the far field, the second SLM has to be programmed with the phase of the same multiple-charged OV. All OV cores at SLM2 are precisely aligned. Decaying after reflection, they repel and redistribute, which does not mean that they overlap initially with the OVs created by SLM1. Nevertheless, the data shown further in this paper clearly confirm, that this “non-local” erasure of the TCs happens (actually strong attraction between and annihilation of oppositely-charged OVs) providing the suitable condition for GBB generation in the focal plane of the focusing lens L and further behind it. Please note that the lens *does not* perform near-field imaging of the generated ring from SLM1. It is acting after SLM2 as a Fourier transform element to generate the GBB in the artificial far field.

### Zeroth-order Gauss-Bessel beam generation

**Figure 3:**
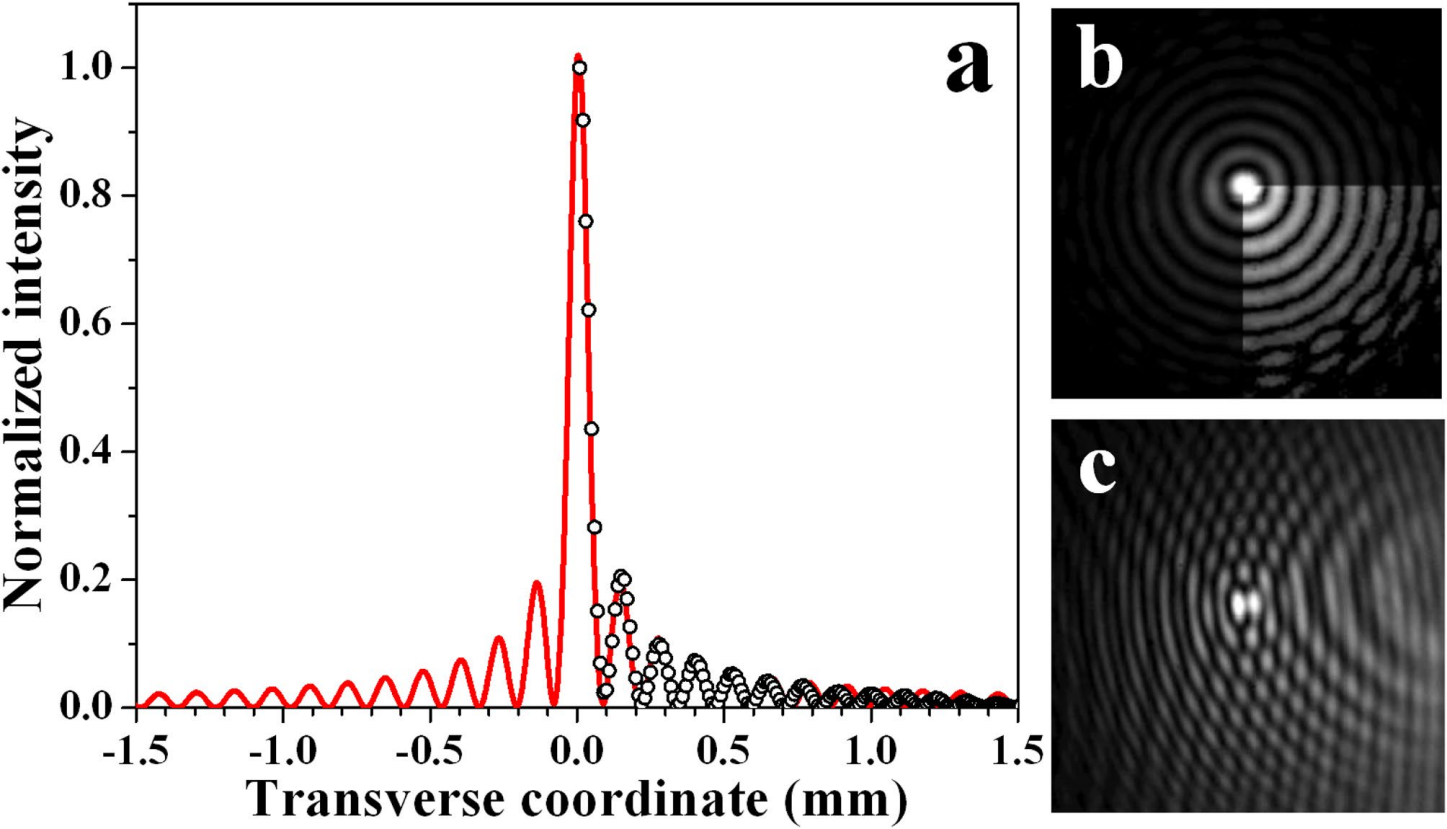
**(a)** Radial intensity profile of the zeroth-order GBB (hollow circles) generated by annihilating OV with a $|TC|=21$ ($r_0/\omega _0=15.0$ in the plane of the lens) and the respective numerically generated transverse beam cross-section (red solid curve). Power density distribution of this GBB **(b)** and interference pattern **(c)** obtained by overlapping it with an offset spherical wave. The CCD camera is located 15 cm (4 Rayleigh diffraction lengths $z_R$ of the focused pure Gaussian beam) behind the focus of the lens.

In Fig. 3(a), the circles show the radial intensity profile of the zeroth-order Gauss–Bessel beam generated by Fourier-transforming bright ring with ring-to-width ratio $r_0/\omega _0=15$ (annihilating an OV with $|TC|=21$). This profile is extracted from the power density distribution shown in frame (b). The numerically generated fit of a Bessel beam corresponding to the experimental data is presented in the graph with a red solid curve. As seen, both the positions of the central peak and of the first five satellites and their relative intensities match very well with the experimental points. However, as it should be expected for a GBB with a finite energy (see Eq. (10)), the intensities of the satellites lying further off axis decrease more rapidly as compared to the theoretical Bessel curve. One quarter sector of the frame in Fig. 3(b) is adjusted in brightness in order to more easily recognize the presence of weaker, farther lying rings and the fact that some of them are azimuthally modulated and even broken. The latter is probably due to the non-perfect initial conditions in front of the focusing spherical lens, which performs a two-dimensional Fourier transformation in space, specifically penetration of some light in the central dark (but not entirely black) area in the middle of the bright OV ring (see the interference pattern in Fig. 2(a)), and eventually, some aberrations of the bright OV ring at large radius-to-width ratio $r_0/\omega _0$. Because of these somewhat modulated and even broken farther-lying rings of the Gauss-Bessel beams, the experimental results (circles) in the graph in Fig. 3 are showing azimuthally-averaged data. The interference pattern shown in Fig. 3(c) is obtained with the beam presented in frame (b) when overlapping it with an offset spherical wave. In this particular case, OVs with TCs $=21$ and $-21$ are annihilated achieving $r_0/\omega _0=15$ in the plane of the focusing lens. From this interferogram it is evident that there is no fork-like splitting of the interference line crossing the central peak of the GBB. Hence, we can consider the beam as a zeroth-order Gauss–Bessel beam. Second, one can recognize that the interference lines crossing each ring are offset at a half of their period with respect to these crossing the neighboring rings. The presence of such phase jumps of $\pi$ is in agreement with the data in Ref.^7^ and results in a perfect destructive interference of the signal between the satellite rings of the GBB down to the noise level.

**Figure 4:**
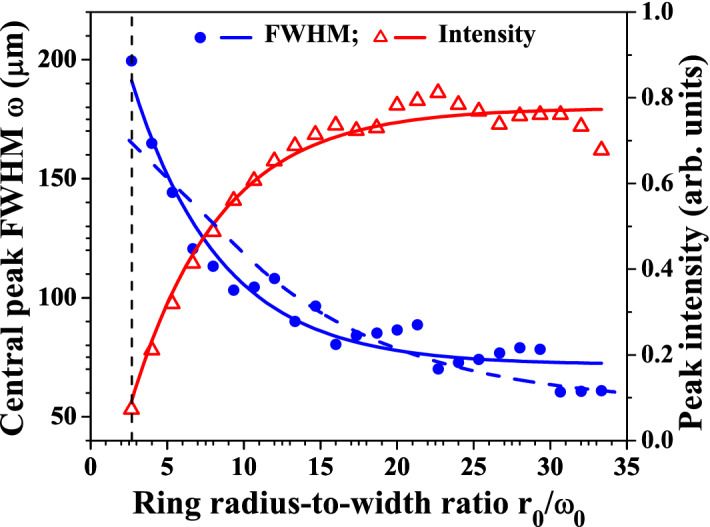
Blue dots/solid line and left scale: Experimental dependence of the full width at half maximum (FWHM) of the central peak of the zeroth-order GBB on the radius-to-width ratio $r_0/\omega _0$ of the bright ring measured in the plane of the lens. Blue dashed curve - numerical result obtained using Eq. (10). Red triangles/solid line and right scale: Central peak intensity vs. $r_0/\omega _0$ extracted from the same set of experimental data. The CCD camera is located 45 cm (12 Rayleigh diffraction lengths $z_R$ of the focused pure Gaussian beam) behind the focus of the lens.

Figure 4 shows the influence of the ring radius-to-width ratio $r_0/\omega _0$ in the plane of the focusing lens on the width of the central peak (blue solid line/solid circles; left ordinate) and on its peak intensity (red line/open triangles; right ordinate) at a fixed longitudinal position $z=45\,cm$ ($z=11.8z_R$; $z_R$—Rayleigh diffraction length of the focused pure Gaussian beam) behind the focus of the lens. The blue dashed curve in Fig. 4 shows a numerical result for the central peak (FWHM) obtained using Eq. (10), which is in qualitative agreement with the experimental data. The strong dependencies are no surprise, since the absolute value of the TC (encoded and, later, annihilated) is playing a crucial role in forming the bright vortex ring in front of the focusing lens. Up to $r_0/\omega _0=20-25$, the experimentally observed general tendency shown in Fig. 4 is that, the higher $r_0/\omega _0$, the higher the peak intensity and the lower its transverse width. At even higher $r_0/\omega _0$ the dependence seems to saturate. The detailed inspection of the experimental data shows that at $r_0/\omega _0>25$ (i.e. at annihilated $|TC|>35$) the weak decrease in the central peak intensity is accompanied by an increase of the number of well formed modulation-free outer-lying rings of the GBB (see Fig. 6(c,d)).

**Figure 5:**
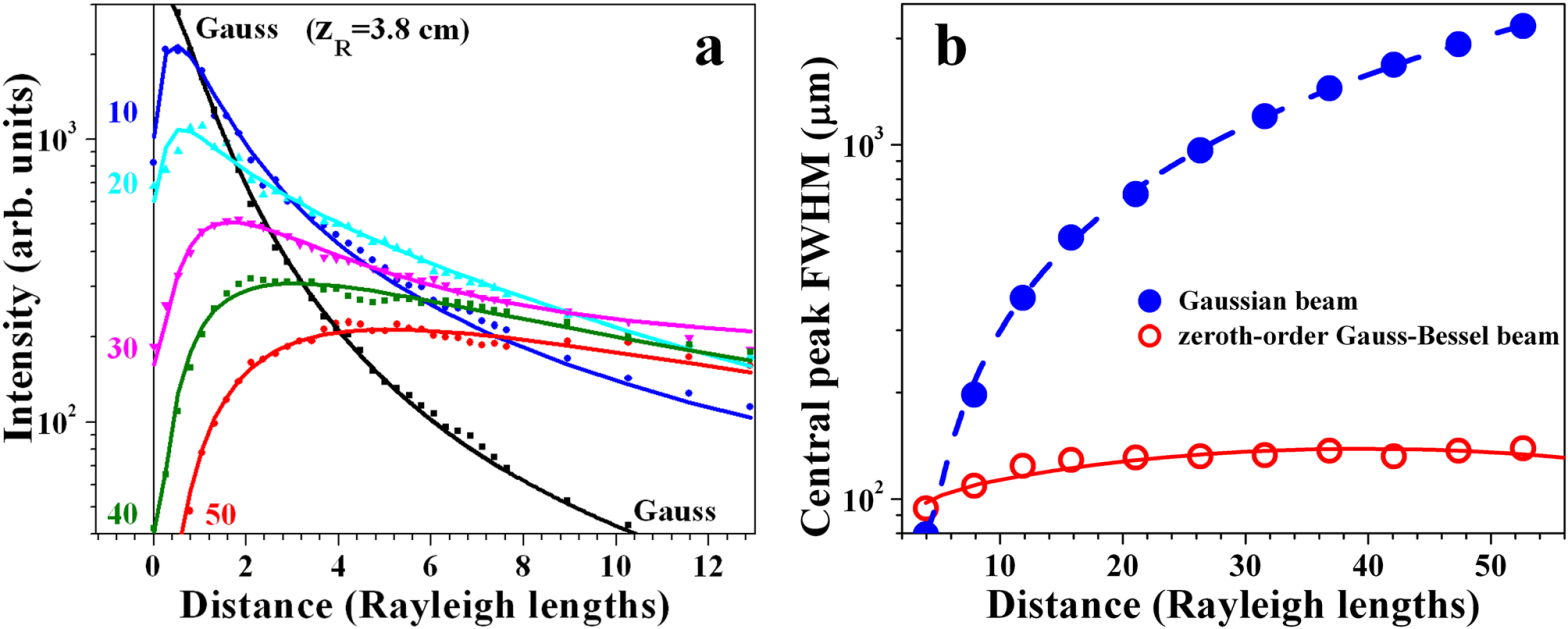
Graph **(a)**—intensity of the Gaussian beam vs. propagation distance behind the focus of the lens (black curve) in units of Rayleigh diffraction length ($z_R$=3.8 cm). For comparison, the variation of the central peak intensity of the Gauss-Bessel beams vs. distance (in units of $z_R$) are shown for |*TC*|=10, 20, 30, 40, and 50 corresponding to $r_0/\omega _0$= 8.7, 14.8, 21.2, 29.2, and 31.6 (color lines). Graph **(b)**—measured width (FWHM) of a Gaussian beam (blue solid dots) and of the central peak of the Gauss–Bessel beam (red hollow circles) vs. free space propagation distance (in units of $z_R$, in the particular measurement—from 15 to 200 cm behind the focus of the lens). The GBB is generated using OVs with topological charges $|TC|=30$ on the SLMs ensuring $r_0/\omega _0$=21.2 in the plane of the lens.

In Fig. 5 we present data which, in view of potential applications of long-range GBBs generated by annihilating OVs, seem to be the most important. In Fig. 5(a) we compared the intensity of the Gaussian beam vs. propagation distance behind the focus of the lens (black curve) with the variations of the central peak intensities of the Gauss–Bessel beams vs. distance for $r_0/\omega _0=$ 8.7, 14.8, 21.2, 29.2, and 31.6 (corresponding to |*TC*|=10, 20, 30, 40, and 50; color lines). The rapid decrease of the intensity of the Gaussian beam vs. propagation length is expected. The evolution of the intensity of the beam generated at $r_0/\omega _0=$ 8.7 (although this value is at the limit of the validity of our model) already shows the main features of all curves - initial increase of the peak intensity (in this case around $z=z_R$) followed by a monotonic decrease. Interestingly, the higher the ring radius-to-width ratio $r_0/\omega _0$, the closer the curves at higher propagation distances. The central peaks of the Gaussian and of the Gauss-Bessel beams (Fig. 2(b)) were followed by translating the CCD camera up to two meters behind the focus of the lens (i.e. up to 53 Rayleigh diffraction lengths $z_R$ of the Gaussian beam). The CCD sensor is kept directly illuminated. The long-range non-diffracting nature of the generated beam is clear from the red open circles and solid lines in Fig. 5(b). As expected and already mentioned, the pure Gaussian beam focused by the lens L is broadening drastically. To this end, both SLMs are simply switched off such that they act as mirrors. The contrast to the zeroth-order Gauss-Bessel beam is evident and impressive in the used logarithmic scale. We stopped the scan at $z=53z_R$ (2 meters behind the focus) in order to keep the data for the Gaussian beam valid, which, at large distances, were extracted from interpolating its central parabolic profile recorded directly by illuminating the chip of the CCD camera. In our view the comparison fully justifies to speak of long-range GBBs with divergence on the microradian scale.

**Figure 6:**
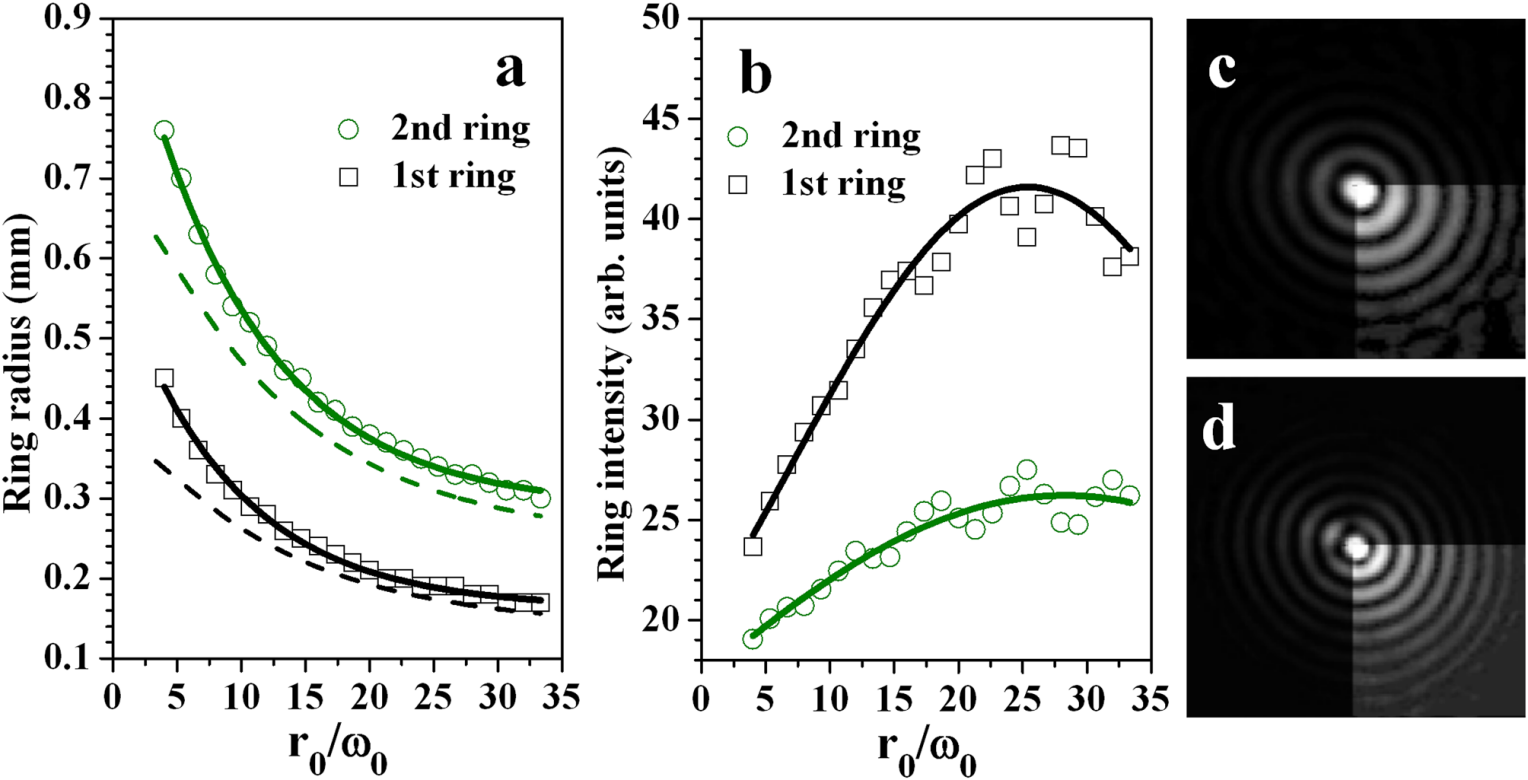
Decrease of the radii of the 1st and 2nd ring of the zeroth-order GBB **(a)** vs. $r_0/\omega _0$. Dashed curves - numerical results obtained using Eq. (10). Respective increase of the peak intensities **(b)** with increasing the bright ring radius-to-width ratio $r_0/\omega _0$ in the plane of the focusing lens. Frames **(c,d)**—GBBs generated in the cases $r_0/\omega _0=$14.8 and 29.2 (using OVs with $|TC|=20$ and 40) indicating that the number of unperturbed rings increases with $r_0/\omega _0$. The data are recorded 45 cm (12$z_R$) behind the focus of the lens.

We already commented on the strong influence of the magnitude of the ring radius-to-width ratio on the parameters of the central peak – width and peak intensity (see Fig. 4). It is natural to expect that the neighboring rings of the Gauss-Bessel beam will also be influenced from $r_0/\omega _0$. Since the central peak width decreases with increasing $r_0/\omega _0$, it can be expected that the neighboring rings will also shrink radially at higher $r_0/\omega _0$. Indeed, this tendency is observed both experimentally and numerically and is presented in Fig. 6(a) for the first and second ring of the zeroth-order GBB. It is in agreement with the theoretical results shown in Fig. 1(c) and readily explained by the fact that the larger $r_0/\omega _0$ due to larger (later annihilated) TCs, the smaller the satellite bright ring radius. The dashed curves in Fig. 6(a) obtained numerically using Eq. (10) qualitatively well reproduce the experimental tendencies. The narrowing of the central peak when the $r_0/\omega _0$ is increased is accompanied by an increase of the central peak intensity up to $r_0/\omega _0=$25 (see Fig. 4). A similar behavior can be expected when considering the first and second rings of the GBB (see Fig. 6(b)). In both graphs in Fig. 6 the solid fit curve is intended to guide the eye only. As mentioned, at values of $r_0/\omega _0>30$ (annihilating TCs exceeding approximately 40), the nearly 8% reduction of the central peak intensity is accompanied with an increase in the number of the rings with no azimuthal modulation. Because of the energy conservation, the observed decrease of the intensities of the central peak and of the first two central rings of the GBB leads to an energy redistribution towards the outer lying rings which become better visible. The experimental data confirming this expectation are shown in panels (c) and (d) of Fig. 6 for $r_0/\omega _0=$14.8 and 29.2, respectively (annihilating OVs with TCs equal to |*TC*|=20 and 40).

### First-order Gauss–Bessel beam generation

**Figure 7:**
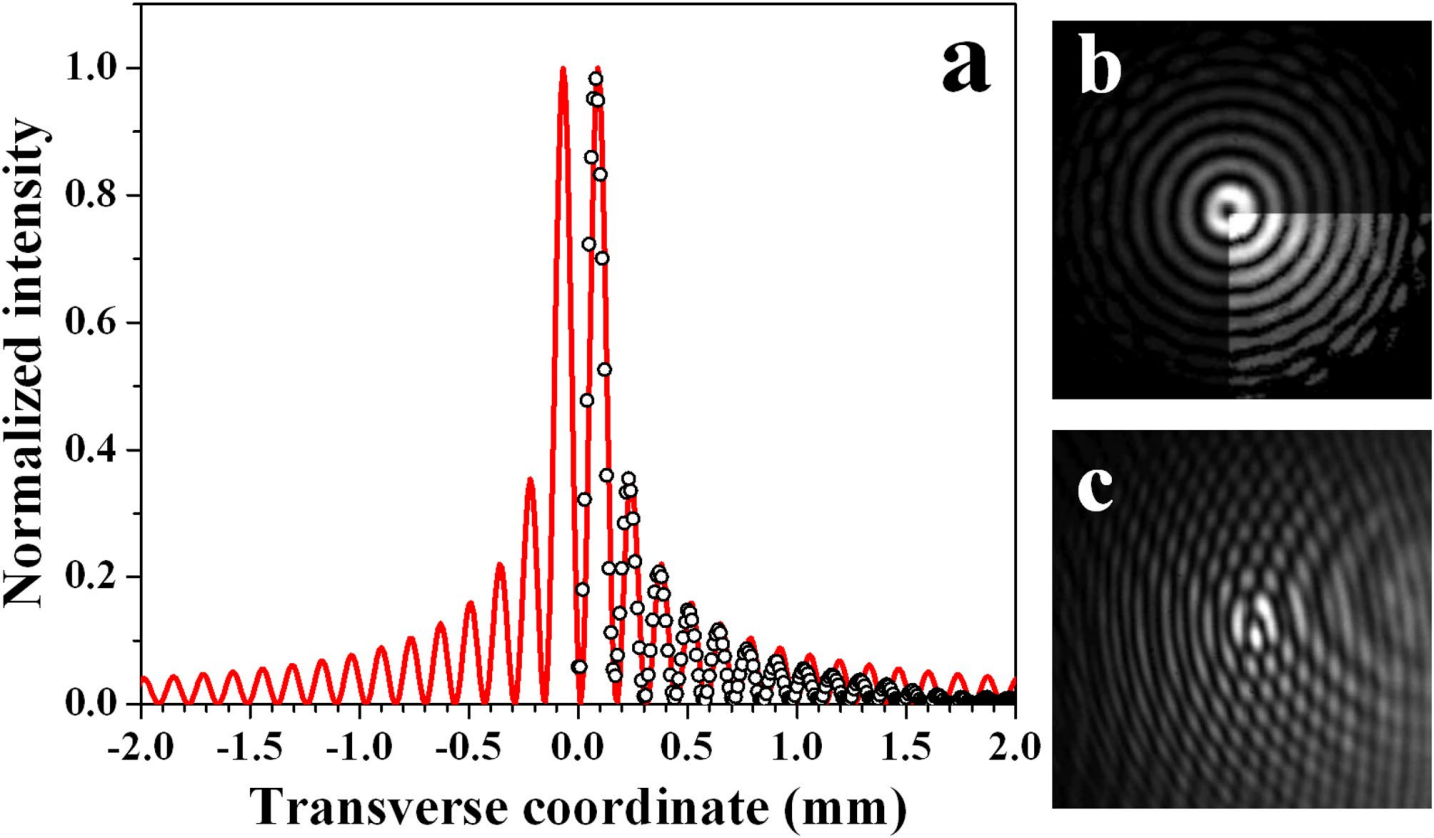
**(a)** Radial intensity profile of the first-order Gauss-Bessel beam (hollow circles) generated by annihilating 20 from the total 21 OVs ($r_0/\omega _0=15$ in the plane of the lens) and numerical fit (red solid curve). Power density distribution of this GBB **(b)** for the same $r_0/\omega _0$ and interference pattern **(c)** obtained by overlapping it with an offset spherical wave. The data are recorded 15 cm (4$z_R$) behind the focus of the lens.

In Fig. 7, following the style of presentation of Fig. 3, we show the radial intensity profile of a first-order Gauss–Bessel beam (circles) generated by Fourier-transforming bright ring-shaped beam with radius-to-width ratio $r_0/\omega _0=15$ and unit residual topological charge. This beam is created by programming an OV with a TC=21 on SLM1 and annihilating 20 TCs after reflecting the beam from SLM2. The profile is extracted from the power density distribution shown in Fig. 7(b). The numerical fit with a first-order Bessel beam is presented in the graph with a red solid curve. As in the case of the zeroth-order GBB, the positions of the first six concentric rings and their relative intensities match the experimental points very well. However, as expected for a GBB with a finite energy, the intensities of the farther outside lying satellites decrease more rapidly in the experiment as compared to the theoretical curve. Again, one quadrant of the frame of Fig. 7(b) is adjusted in brightness in order to ease the recognizability of the presence of weaker, remote rings.

By increasing the propagation distance behind the lens focus (up to 2.5 m in some measurements, equivalent to 65 Rayleigh diffraction lengths of the focused Gaussian beam), we clearly observed a weak decrease of the intensity of the central ring. However, the observed absence of its broadening results in forming a larger number of concentric rings with no azimuthal intensity modulation. This will be discussed later in this paper.

In Fig. 7(c), analogous to Fig. 3(c) for the zeroth-order GBB, we show the interference pattern created by superimposing the first-order GBB with an offset spherical wave. In this particular case, 20 of the encoded 21 OVs are annihilated. A downward fork-like splitting of one single interference line in the center of Fig. 7(c) is evident, thus confirming that the generated wave is a first-order Gauss–Bessel beam with an on-axis point phase dislocation. Inspecting the remote rings, one can recognize that the interference lines crossing adjacent rings are offset by half of their period. This is a clear indication for the presence of radial $\pi$-phase jumps between them and explains the observed decrease of the signal between the satellite rings of the first-order GBB down to the noise level.

**Figure 8:**
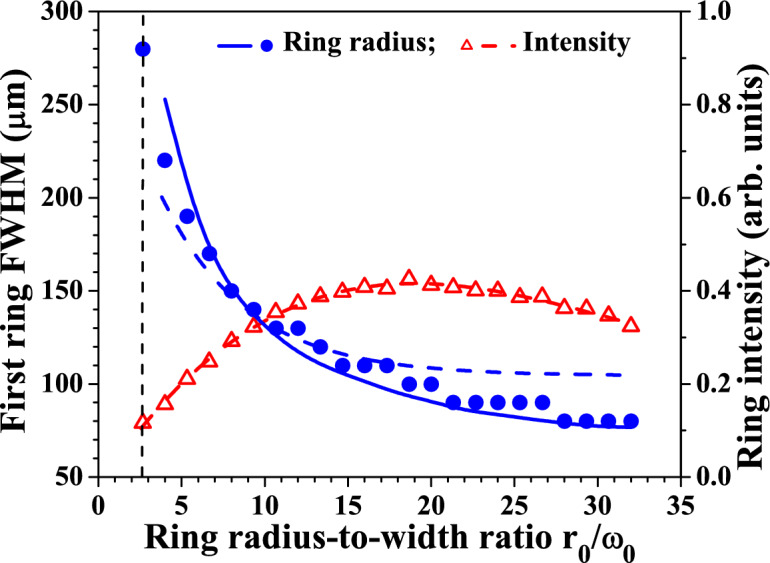
Blue dots/solid line and left scale: Dependence of the first ring radius (FWHM) of the first-order GBB on the $r_0/\omega _0$. (One OV remains nested in the beam causing its central part to be doughnut-shaped.) Blue dashed curve—numerical result obtained using Eq. (10). Red triangles/dashed line and right scale: Azimuthally-averaged first ring intensity vs. $r_0/\omega _0$ extracted from the same set of data. The data are recorded 45 cm (12$z_R$) behind the focus of the lens.

As for the zeroth-order Gauss-Bessel beams, one can expect that there are well pronounced dependencies of the ring radii and ring peak intensities of the first-order GBB on the ring radius-to-width ratio $r_0/\omega _0$ of the beam in front of the focusing lens (i.e. on the number of erased vortices in front of the lens). The dependence of the peak intensity on $r_0/\omega _0$ is shown in Fig. 8 using red triangles and a dashed line referring to the right ordinate. The dependency exhibits a well-formed maximum for $r_0/\omega _0 \approx 20$ followed by a decrease at larger values. Well pronounced is also the tendency that the higher $r_0/\omega _0$, the lower the transverse width of the central ring of the GBB (Fig. 8, blue dots/solid line and left ordinate). The blue dashed curve in Fig. 8 is a numerical result obtained using Eq. (10). As seen, the theoretical tendency is in a qualitative agreement with the experimental result.

**Figure 9:**
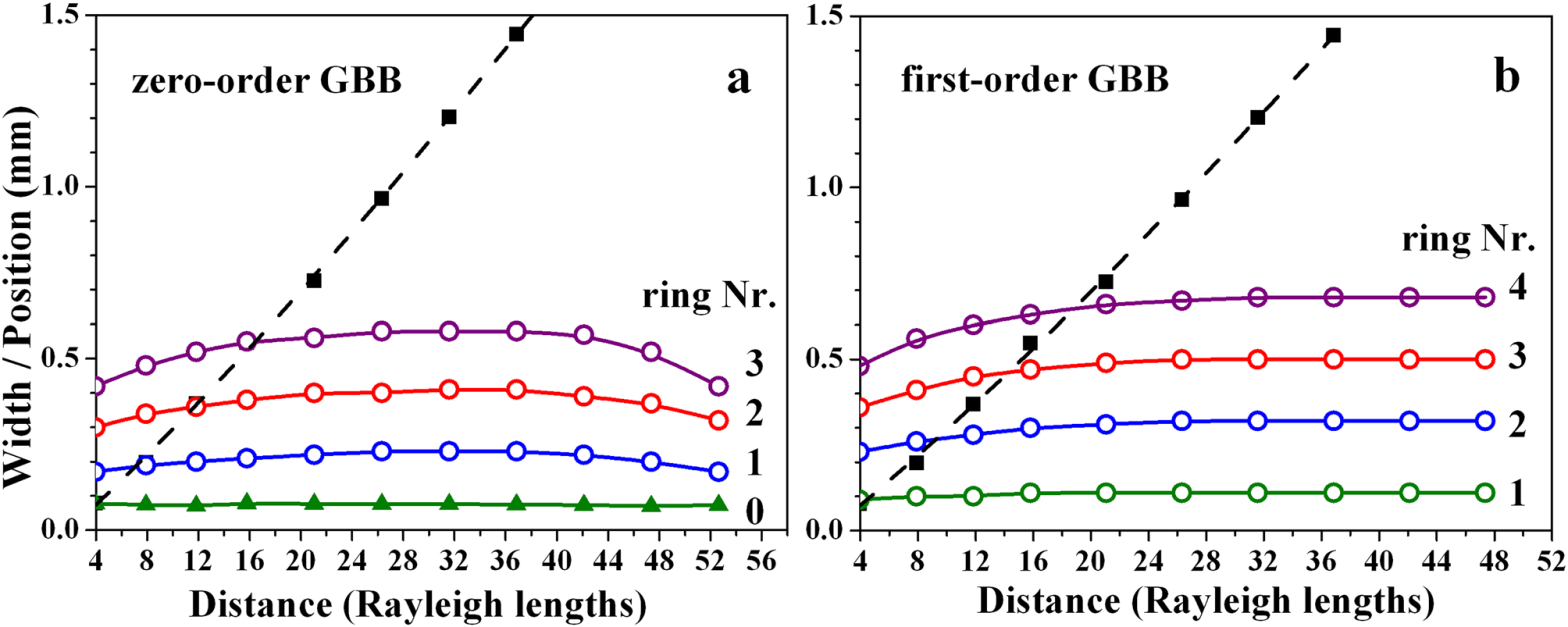
Direct comparison between the width of the central peak for zeroth-order GBB and the positions of the first coaxial bright rings (graph **(a)**) and respective data for first-order GBB (graph **(b)**) vs. free space propagation distance in units of Rayleigh lengths ($z_R=$3.8 cm). Black solid squares and dashed line-width of the pure Gaussian beam when the two SLMs are switched off.

In Fig. 9, we present the width (FWHM) of central peak of the zeroth-order GBB, as well as the dependencies of the radii of the first three (four) rings for a zeroth- and first-order GBB for propagation distances up to 2 m ($z=52.6z_R$). In this particular comparison $r_0/\omega _0=21.2$ for zeroth-order GBB and $r_0/\omega _0=21.4$ for first-order GBB carrying residual unit topological charge. For comparison, the experimental data obtained for the width (FWHM) of a pure Gaussian beam (when both SLMs are switched off) are shown with black solid squares and black dashed lines. In our view, this result, along with the data presented in Fig. 5, fully justifies the characterization of the generated zeroth- and first-order Gauss-Bessel beams as long range Gauss-Bessel beams.

**Figure 10:**
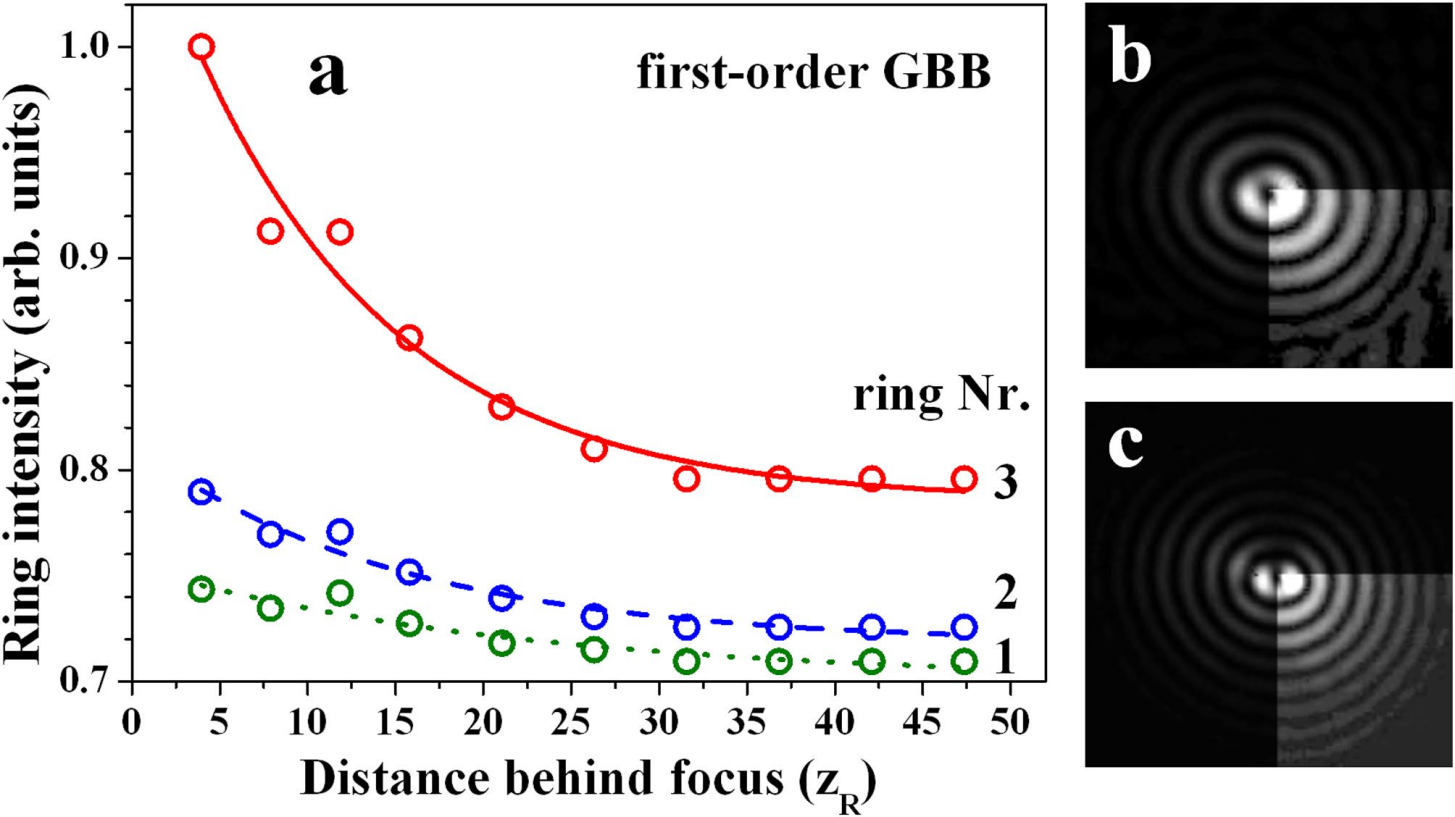
**(a)** Azimuthally-averaged peak intensity of the first three rings of the first-order GBB for $r_0/\omega _0=$21.4 (annihilating OVs with $TC=30,-31$). (b,c) first-order GBBs generated using $TC=20,-21$ and $TC=40,-41$ ($r_0/\omega _0=$ 15.0 and 30.0, respectively), indicating that the number of unperturbed rings increases with $r_0/\omega _0$ and with |*TC*|.

In Fig. 10 we present the evolution of the peak intensities of the first three rings of a first-order GBB. The data refer to $r_0/\omega _0=21.4$ (encoded TCs 30 and -31 since one OV remains in the resulting beam). The decrease of the peak intensities of the rings is evident when the propagation distance increases. As seen in Fig. 9(b) the ring positions do not change significantly. The modulation depth also does not decrease (not shown here). The explanation for the energy conservation is in the increased transfer of power to larger number of outer lying rings with less and less pronounced azimuthal modulation. This is demonstrated in frames (b) and (c) in Fig. 10 for $r_0/\omega _0= 15$ and 30 recorded at $z=11.8z_R$. Qualitatively, one can state that at increased propagation distances the first-order Gauss–Bessel beam generated by this technique is also more and more evolving into a better finite-energy approximation of a perfect first-order Bessel beam.

## Conclusion

In conclusion, we experimentally demonstrated that zeroth- and first-order long-range non-diffracting Gauss-Bessel beams can be created by initially generating and subsequently annihilating multiple-charged optical vortices. The developed analytical model is supporting the experimental results. The observed general tendencies can be summarized as follows. (i) The long-range Gauss-Bessel beams have negligible transverse evolution up to 2 m corresponding to free-space propagation distance of approximately 53 Rayleigh diffraction lengths of the focused pure Gaussian beam. In view of their divergence of a few microradians they can be regarded as non-diffracting. (ii) The increase of the ring radius-to-width ratio of the beam in the plane of the focusing lens, obtained by increasing the subsequently annihilated topological charges, leads to a radial shrinking of the peak and the oscillating beam wings. (iii) The central peak/ring intensities reach maximal values when the beam’s radius-to-width ratio $r_0/\omega _0$ in the plane of the focusing lens is increased to 20–25. (iv) Even larger values of $r_0/\omega _0$ lead to lower intensities. (v) At a fixed value of $r_0/\omega _0$, i.e. at fixed value of |*TC*|, the decrease of the peak intensity at larger propagation distances is due to power/energy redistribution towards the outer lying rings. Qualitatively, as the free-space propagation behind the focus of the lens is increased, the analyzed zeroth- and first-order Gauss-Bessel beams are more and more evolving in to better finite-energy approximations of the perfect zeroth- and first-order Bessel beam. The used experimental setup can easily be modified such that only one spatial light modulator is required. In this case, the two desired phase distributions would be encoded on respectively one half. The approach does not require any special optical elements and is accessible in many laboratories equipped with spatial light modulators. Unfortunately, SLMs cannot survive high pulse energies/intensities. In such cases digital micromirror devices or reflective etched vortex phase plates could be used.

## Supplementary information

Supplementary Figures.

## Data Availability

The datasets generated during and analyzed during the current study are available from the corresponding author on reasonable request.
