# Supplementary material for: Zeroth- and first-order long range non-diffracting Gauss–Bessel beams generated by annihilating multiple-charged optical vortices
Source: Sci Rep. 2020 Dec 15;10:21981. doi: 10.1038/s41598-020-78613-7 (PMC7738530; doi:10.1038/s41598-020-78613-7)
Supplement: Supplementary file 1 — Supplementary Figures. [file 41598_2020_78613_MOESM1_ESM.pdf]

Supplementary material to the manuscript entitled

Zeroth- and first-order long range non-diffracting Gauss-Bessel beams generated by annihilating multiple-charged optical vortices

Lyubomir Stoyanov[1], Maya Zhekova[1], Aleksander Stefanov[2,3], Ivan Stefanov[1], Gerhard G. Paulus[4,5], and Alexander Dreischuh[1,*]

[1]Department of Quantum Electronics, Faculty of Physics, Sofia University, 5, J. Bourchier Blvd., Sofia-1164, Bulgaria
[2]Department of Mechatronics, Robotics and Mechanics, Faculty of Mathematics and Informatics, Sofia University, 3,J. Bourchier Blvd., Sofia-1164, Bulgaria
[3]Institute of Mathematics and Informatics, Bulgarian Academy of Sciences, Acad. Georgi Bonchev Str., Block 8, Sofia-1113, Bulgaria
[4]Institute of Optics and Quantum Electronics, Friedrich Schiller University, Max-Wien-Platz 1, D-07743 Jena,Germany
[5]Helmholtz Institute Jena, Helmholtzweg 4, D-07743 Jena, Germany

*ald@phys.uni-sofia.bg

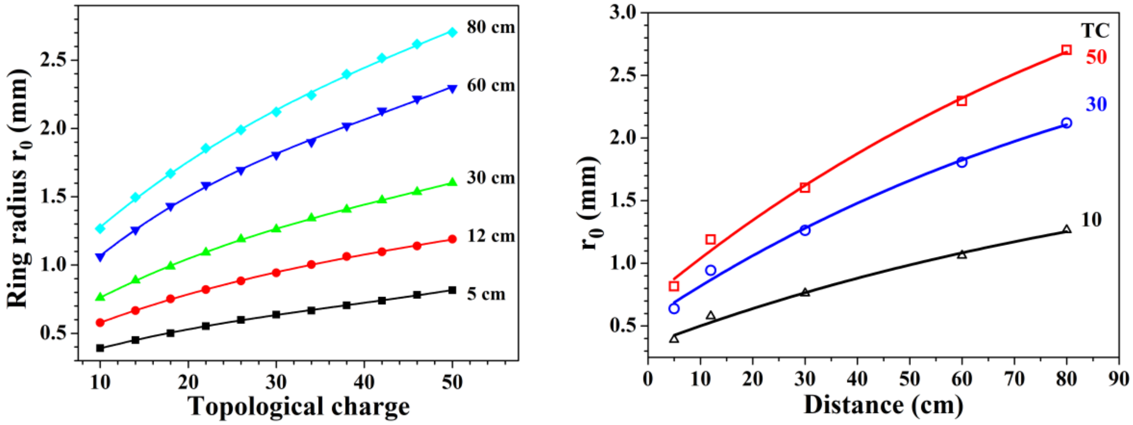

Figure S1: Left: Vortex ring radius r_0 vs. encoded topological charge for different distances after the used SLM. **Right:** Ring radius r_0 as function of the propagation distance for optical vortices with TCs=10, 30, and 50.

===================

Measured central peak intensity and intensities of the 1st and 2nd neighboring rings as a function of the propagation distance behind the focus of the lens for r_0/ω_0=21.2 and 31.6 (initially created and subsequently annihilated TCs 30 and 50). The data show similar tendencies in the dependence intensity vs. distance – maximum at some 30 cm (z=8z_R) behind the focus followed by a monotonic decrease. The relative intensities of the 1st ring to the central peak and the 2nd ring to the central peak vs. distance behind focus increase from 17.5% to 24% for the 1st ring and from 10% to 15% for the 2nd ring. This means that the energy of the GBB is slowly redistributing towards the outer lying rings (see frames (c) and (d) in Fig. 6 in the manuscript), i.e. the Gauss-Bessel beam is evolving more and more in to a better finite-energy approximation of a perfect Bessel beam.

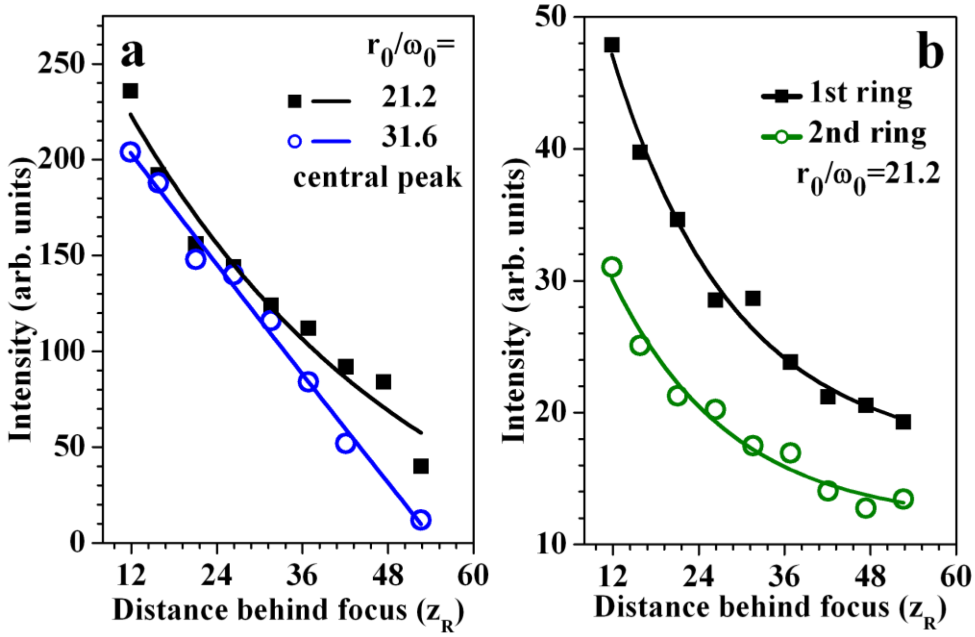

Figure S2:(a) Intensity of the central peak of the zeroth-order GBB vs. distance behind the lens focus for r_0/ω_0=21.2 and 31.6 in the plane of the lens (OV |TC| = 30 and 50 on the SLMs). **(b)** Azimuthally-averaged peak intensity of the 1st and 2nd ring of the GBBs generated with OVs with r_0/ω_0=21.2.

If a low angle axicon (opening angle γ = 10 mrad; refractive index n=1.5; opening angle of the cone $\theta = (n-1)\gamma$ = 5 mrad) and an input Gaussian beam with half width at half maximum ω_0=1 cm are used, the estimated maximal propagation distance of the GBB is z_{max}=2 m (see e.g. Eqs. 9 and 10 in Ref. [4] in the main text). We calculated the phase of such an axicon and used it to program SLM2 removing the lens L and keeping the rest of the experimental setup unchanged. The cross-sections of the GBB generated by this axicon phase are compared with the cross-sections of the GBB generated by our method at four propagation distances. The data shown in Fig. S3 refer to ring radius-to-width ratio r_0/ω_0=21.2 in front of the focusing lens (due to initially generated and subsequently annihilated topological charges |TC|=30). As evident from panels (a) and (b) of this figure, at $z=z_R$=7.9 and 23.7 the central peaks' widths of the pairs of GBBs are equal. However, in the case of a simulated axicon, the number of the outer-lying rings is less. At propagation distances higher than z=150 cm =39.5z_R, the modulation depth decreases and the beam obtained with the axicon distribution is not a Bessel beam anymore. In contrast, the GBBs generated by annihilating OV with high TC retain their high quality.

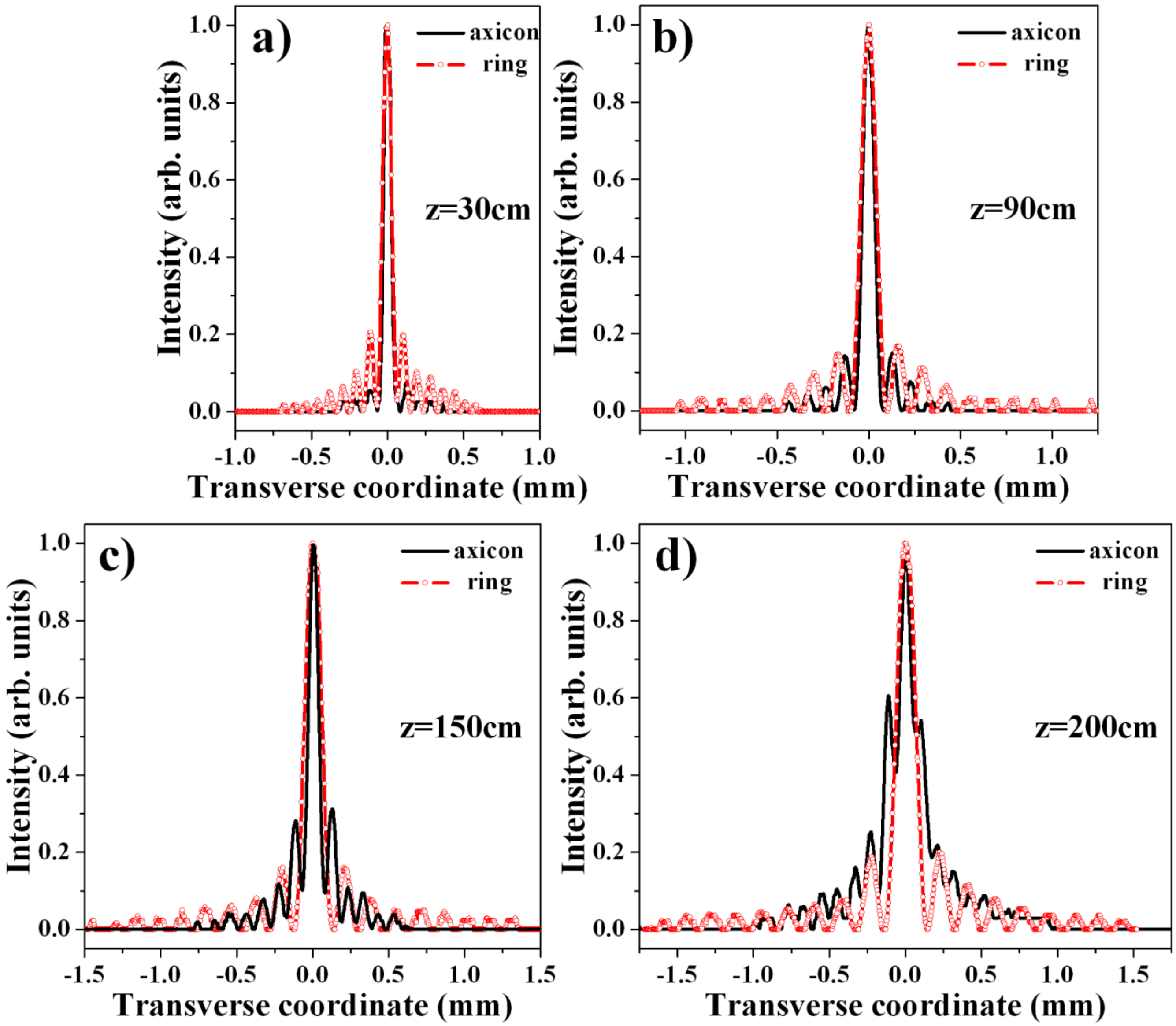

Figure S3: Radial intensity profiles of zeroth-order GBBs generated by simulated axicon phase (opening angle γ=10 mrad; refractive index n=1.5; opening angle of the cone $\theta = (n\text{-}1)\,\gamma$ = 5 mrad; black solid curves) and by annihilating OV with a |TC| = 30 (red curves and open circles; r_0/ω_0=21.2 in front of the focusing lens) at propagation distances 30 cm=7.9z_R (a), 90 cm=23.7z_R (b), 150 cm=39.5z_R (c), and 200 cm=52.6z_R (d).

In Fig. S4 we provide additional evidence for this behavior when r_0/ω_0=29.2. This case corresponds to OVs with |TC|=40, initially created (to form the required bright ring-shaped beam) and subsequently annihilated (to ensure the flat phase profile in front of the focusing lens, which is a necessary condition to create zeroth-order GBB).

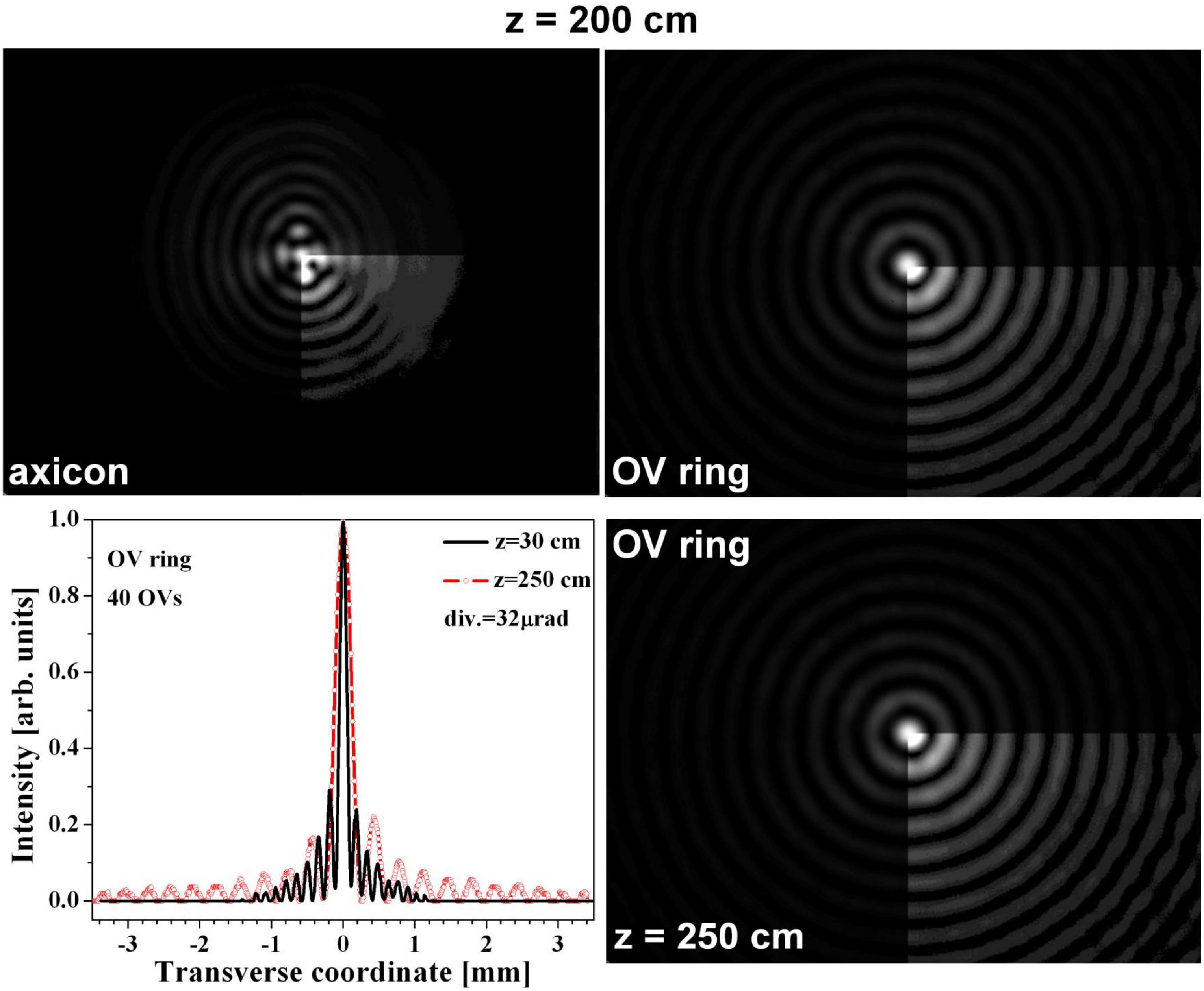

Figure S4: Comparison between Gauss-Bessel beams (GBBs) created by simulated axicon (**upper left panel**) and by initially creating and subsequently annihilating OVs with topological charges |TC|=40 (r_0/ω_0=29.2) (**upper right panel**) at a propagation distance of 200 cm behind the focusing lens. **Graph:** Cross-sections of GBBs generated by the method described in the manuscript at propagation distances z=30 cm and 250 cm (**lower right panel**) after the focus of the lens. The data confirm the denoted divergence in the microradian scale.
